# Endoscopic vacuum therapy for the treatment of colorectal leaks — a systematic review and meta-analysis

**DOI:** 10.1007/s00384-021-04066-7

**Published:** 2021-11-24

**Authors:** Florian Kühn, Josefine Schardey, Ulrich Wirth, Tobias Schiergens, Alexander Crispin, Nicola Beger, Dorian Andrade, Moritz Drefs, Petra Zimmermann, Maria Burian, Joachim Andrassy, Jens Werner

**Affiliations:** 1 Department of General, Visceral, and Transplant Surgery, Ludwig-Maximilians-University Hospital - Campus Grosshadern, Marchioninistr. 15, 81377 Munich, Germany; 2 Institute for Medical Information Processing, Biometry, and Epidemiology (IBE), Ludwig-Maximilians-University Munich, 81377 Munich, Germany

**Keywords:** Endoscopic vacuum therapy, EVT, Colorectal defects, Anastomotic leakage, Rectal stump leakage

## Abstract

**Background:**

During the last two decades, vacuum-assisted wound therapy has been successfully transferred to an endoscopic treatment approach of various upper and lower gastrointestinal leaks called endoscopic vacuum therapy (EVT). As mostly small case series are published in this field, the aim of our systematic review and meta-analysis was to evaluate the efficacy and safety of EVT in the treatment of colorectal leaks.

**Methods:**

A systematic search of MEDLINE/PubMed and Cochrane databases was performed using search terms related to EVT and colorectal defects (anastomotic leakage, rectal stump insufficiency) according to the PRISMA guidelines. Randomized controlled trials (RCTs), observational studies, and case series published by December 2020 were eligible for inclusion. A meta-analysis was conducted on the success of EVT, stoma reversal rate after EVT as well as procedure-related complications. Statistical interferences were based on pooled estimates from random effects models using DerSimonian-Laird estimator.

**Results:**

Only data from observational studies and case series were available. Twenty-four studies reporting on 690 patients with colorectal defects undergoing EVT were included. The mean rate of success was 81.4% (95% CI: 74.0%–87.1%). The proportion of diverted patients was 76.4% (95% CI: 64.9%–85.0%). The mean rate of ostomy reversal across the studies was 66.7% (95% CI: 58.0%–74.4%). Sixty-four patients were reported with EVT-associated complications, the weighted mean complication rate across the studies was 12.1% (95% CI: 9.7%–15.2%).

**Conclusions:**

Current medical evidence on EVT in patients with colorectal leaks lacks high quality data from RCTs. Based on the data available, EVT can be seen as a feasible treatment option with manageable risks for selected patients with colorectal leaks.

## Introduction

Since the first reported data in the late 1990s, vacuum-assisted wound therapy has captured nearly every field of surgery by providing a versatile and easy-to-handle method for complicated wounds [1]. In 2001, Weidenhagen et al. started to implement endoscopic vacuum therapy (EVT) at our institution for complication management of anastomotic leakage (AL) after rectal cancer surgery, as a “minimally invasive method for continuous and effective drainage of perianastomotic abscesses and fistula in the pelvic region in combination with debridement and consecutive mechanical closure of the leakage” [2]. Successful definite treatment in 28 of 29 cases of AL without surgical reintervention had been achieved [2].

In the further course, EVT has been successfully applied for interventional treatment of different defects in the upper and lower gastrointestinal tract [3]. AL and other colorectal defects such as Hartmann stump leaks are associated with serious morbidity and mortality [4]. The incidence of AL in rectal cancer surgery ranges between 6 and 30% with an average of 11%, depending on the height of the anastomosis [4]. Therefore, a relevant number of patients are affected by this serious complication and require the best available treatment option. Although preliminary reports showed promising results for EVT in the treatment of colorectal leaks with high success rates and anastomotic salvage, data determining the superiority of EVT over other forms of conservative treatment in diverted anastomoses is lacking [5, 6].

EVT of colorectal leaks is based on the transanal placement of an open-cell microporous sponge intra luminally at the site of-the AL (or other colorectal defects) or through the leak into a extraluminal perianastomotic abscess cavity using a flexible endoscopy [2]. A negative pressure (“vacuum”) is applied via an evacuation tube fixed to the sponge [7]. The sponge is usually changed every 2–4 days until the infection is cleared, and the defect is closed with granulation tissue as proven by endoscopy [7]. This active drainage of the infectious focus results in a decrease of bacterial contamination, local edema, and secretion and is proven to increase perfusion and induce granulation tissue [7].

The conventional treatment of gastrointestinal defects with surgical reinterventions often results in gastrointestinal discontinuity. Additionally, there are other endoscopic therapeutic options like application of stents or attempts for clip closure with uncertain success rates [3]. Today, EVT has evolved to a standard treatment of postoperative surgical leaks in many—mostly European—countries and surgical departments [7–28]. Yet, evidence on efficacy of EVT in the treatment of colorectal leaks lacks high-quality data from randomized controlled trials (RCT), and only few large observation studies are available [26, 27]. In fact, most data on EVT are based on small case series [8, 17, 21, 28–30]. Previously published systematic reviews analyzed the available data on EVT in colorectal defects with small sample sizes within included studies resulting in analysis of up to *n* = 334 patients in total [5, 31, 32]. Nevertheless, in the past few years, the number of studies has increased, reporting high success and low complication rates [5, 6, 27, 33] including a large retrospective series of *n* = 281 cases with the use of EVT treatment on colorectal defects from our institution including the previously published cases of Weidenhagen et al. [2, 27]. To obtain the best available evidence for EVT in the treatment of colorectal leaks, we performed a systematic review and meta-analysis.

## Materials and methods

### Search strategy

For a systematic review with meta-analysis, no approval by the institutional review board was needed. A systematic literature search was carried out by two independent researchers. All references until December 2020 were potentially eligible for inclusion in the study. The following search strategy was used in PubMed, Embase, and Cochrane Database according to the PRISMA checklist [34]: (((((((((EVT) OR (endoscopic vacuum therapy)) OR (endoscopic negative pressure therapy)) OR (endoluminal vacuum therapy)) OR (vacuum-assisted endoluminal)) OR (endosponge)) OR (Endo-sponge)) OR (vacuum-assisted drainage)) OR (Endo-vac)) OR (Endovac)). Subsequently, the reference lists of articles were searched for further relevant literature. Duplicate articles and conference reports without availability of full-text versions were excluded. The remaining articles were screened and filtered by title and abstract, and ultimately a full-text analysis was performed for final evaluation. The full texts of the remaining studies were reviewed independently by two investigators to verify eligibility, and a third reviewer was consulted in the case of disagreement.

### Eligibility criteria

All comparative and cohort studies evaluating the outcome of EVT in the treatment of colorectal defects were considered eligible for inclusion including treatment for AL after colorectal or coloanal anastomosis as well as rectal stump insufficiency following Hartmann’s procedure.

Previous meta-analyses, reviews, case reports, editorials, and letters were excluded. Articles that did not report the primary outcomes of the present meta-analysis were also excluded. Furthermore, studies were excluded, if they were published in any language other than English.

### Outcome parameters

First, we aimed to assess the success of EVT treatment of colorectal defects. Treatment success was usually defined as granulating closure of the cavity without need for further interventional or surgical procedures. Other outcome parameters were the rate of patients with a fecal diversion during EVT treatment, the rate of EVT-associated morbidity and proportion of reversed ostomies. Furthermore, the treatment duration and number of sponges was addressed.

### Data extraction and management

The following data were collected: author details, country, recruitment period, study design, median follow-up, sample size, positive and negative findings, and methodological quality.

Considering the heterogeneity of reported inclusion criteria of each manuscript and lacking sufficient control cohorts, we aimed to review the literature descriptively without the intention of comparative analysis.

### Assessment of publication bias

**Fig. 1:**
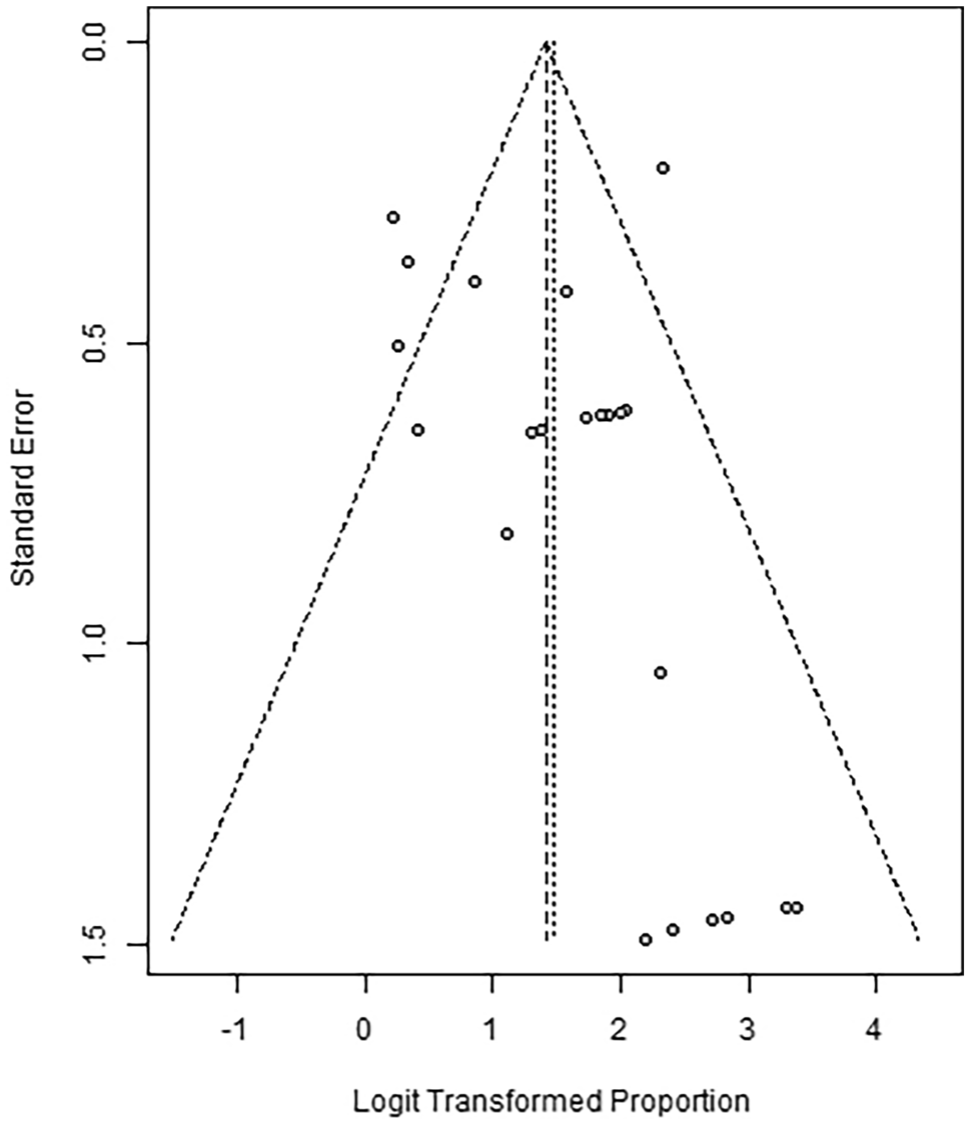
Funnel plot for assessment of publication bias for the EVT success rate

Publication bias across the studies was assessed using funnel plots of the standard error of the success rate of EVT for colorectal defects compared to the success rates in the reporting studies (Fig. 1).

### Statistical analysis

Data were analyzed using meta package version 4.9–1 for R 3.5.0 [35, 36]. We reported pooled rates and their 95% confidence intervals (95% CI) from both fixed effect and random effects models. The former used inverse variance weighting based on the variance estimates for the logit-transformed rates, the latter relied on the DerSimonian-Laird estimators. For assessment of heterogeneity of the study results, we provided tau squared, I squared, and Cochran’s Q tests.

## Results

### Study characteristics

**Fig. 2:**
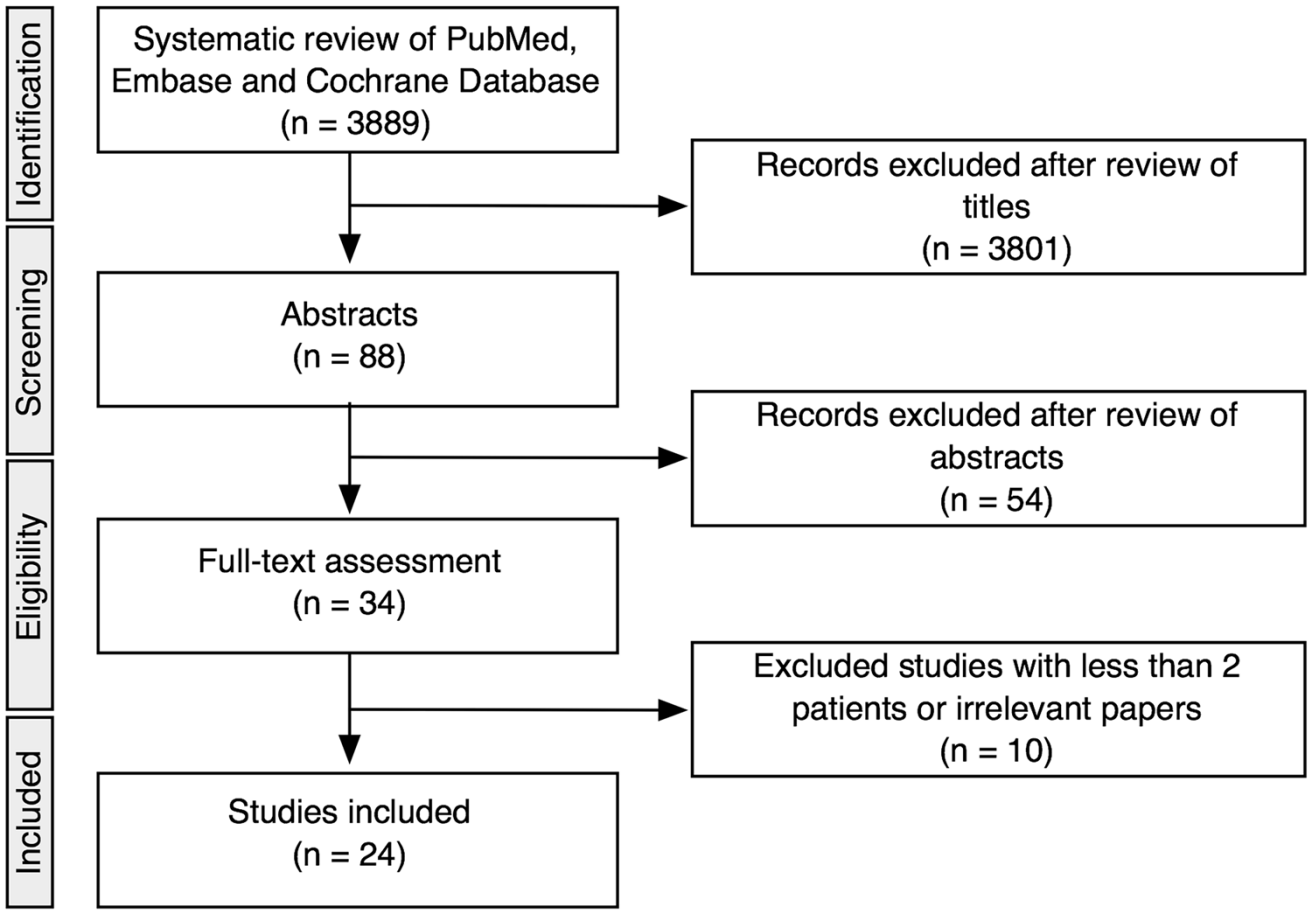
Flow chart of literature selection strategy

After screening 3889 articles by title, 3801 records were excluded. Further 54 studies not related to the current study aims were excluded after a review of the abstracts. Full-text publications underwent authors’ review and assessment of inclusion/exclusion criteria. After full-text assessment of 34 articles, irrelevant studies, or studies with less than 2 patients were excluded. The remaining 24 studies published between 2006 and 2020 were finally selected for analysis (Fig. 2). Any disagreement during the search and selection process was resolved by subsequent consensus.

The characteristics of the included studies are shown in Table 1. The studies were from the following countries of origin: Italy (*n* = 6), Germany (*n* = 5), Netherlands (*n* = 5), Denmark (*n* = 2), Spain (*n* = 1), Sweden (*n* = 1), Austria (*n* = 1), Turkey (*n* = 1), USA (*n* = 1), France (*n* = 1). These publications comprise 18 retrospective studies [3, 8, 9, 12–18, 23–29] and 6 prospective studies [10, 11, 19–22]. Due to the lack of data from RCTs, a few prospective and mostly retrospective case series on EVT treatment of colorectal defects were included in this systematic review.

**Table 1:** Characteristics of the included studies

**Author**	***N***	**Success**	**Diverted patients**	**Reversed stomas**	**Complication rate**	**Mean duration of therapy (d)**	**Mean number of sponge insertions**	**Study character**
Nagell et al. [8]	4	4/4 (100%)	NA	NA	0/4 (0%)	13	NA	R
Mees et al. [28]	5	5/5 (100%)	5/5 (100%)	1/5 (20%)	0/5 (0%)	15	7	R
Van Koperen et al. [9]	16	9/16 (56%)	8/16 (50%)	5/8 (63%)	4/16 (25%)	40	13	R
Von Bernstorff et al. [10]	26	23/26 (88%)	NA	NA	1/26 (8%)	22	6	P
Riss et al. [11]	23	20/23 (87%)	17/23 (74%)	13/17 (76%)	6/23 (26%)	21	NA	P
Verlaan et al. [29]	5	5/5 (100%)	4/5 (80%)	4/4 (100%)	NA	14	3	R
Nerup et al. [12]	13	13/13 (100%)	13/13 (100%)	12/13 (92%)	1/13 (8%)	18	8	R
Srinivasamurthy et al. [13]	8	6/8 (75%)	8/8 (100%)	5/8 (63%)	2/8 (25%)	26	4	R
Arezzo et al. [14]	14	11/14 (79%)	NA	NA	0/14 (0%)	41	13	R
Keskin et al. [15]	15	12/15 (80%)	14/15 (93%)	10/14 (71%)	NA	NA	2	R
Strangio et al. [16]	25	22/25 (88%)	13/25 (52%)	11/13 (85%)	3/25 (12%)	28	9	R
Kuehn et al. [3]	41	34/41 (83%)	19/41 (46%)	15/19 (79%)	4/41 (10%)	20	6	R
Manta et al. [17]	7	7/7 (100%)	NA	NA	0/7 (0%)	NA	NA	R
Mussetto et al. [18]	11	10/11 (91%)	11/11 (100%)	10/11 (91%)	2/11 (18%)	37	16	R
Milito et al. [19]	14	14/14 (100%)	NA	NA	0/14 (0%)	35	NA	P
Borstlap et al. [20]	30	21/30 (70%)	30/30 (100%)	20/30 (67%)	0/30 (0%)	13	4	P
Rottoli et al. [21]	8	8/8 (100%)	8/8 (100%)	7/8 (88%)	NA	12	3	P
Mencio et al. [37]	10	6/10 (60%)	NA	NA	0/10 (0%)	23	7	R
Jimenez-Rodriguez et al.[22]	22	19/22 (86%)	13/22 (59%)	5/13 (38%)	5/22 (23%)	22	3	P
Huisman et al. [23]	20	17/20 88%	18/20 (90%)	14/18 (78%)	NA	25	9	R
Wereen et al. [24]	14	NA	14/14 (100%)	7/14 (50%)	NA	64	19	R
Kantowski et al. [25]	31	18/31 (58%)	13/31 (42%)	5/13 (38%)	6/31 (19%)	21	6	R
Abdalla et al. [26]	47	26/47 (55%)	NA	NA	3/47 (6%)	27	7	R
Kühn et al. [27]	281	256/281 (91%)	221/281 (79%)	132/221 (60%)	27/281 (10%)	25	8	R
**Total**	**690**	566/676	429/568	276/429	64/628			

### Patient numbers and indication for EVT

A total of 690 cases were analyzed. Indications for EVT in this meta-analysis included AL after colorectal surgery, Hartmann stump insufficiency and colorectal perforation (traumatic or in diverticular disease).

### Number of sponge changes and duration of EVT

In 644 out of 690 cases treated with EVT, treatment success was reported after a weighted mean of 6.8 (95% CI: 5.0–9.1, *I*^2^ = 97%, tau^2^ = 0.0701, *p* < 0.01) sponge changes. With 669 of 690 patients reporting the treatment duration, the random effects model showed that the weighted mean of treatment duration was 23.4 days (95% CI: 19.1–28.8, *I*^2^ = 92%, tau^2^ = 0.0331, *p* < 0.01). (Data not shown).

### Success rates of EVT

**Fig. 3:**
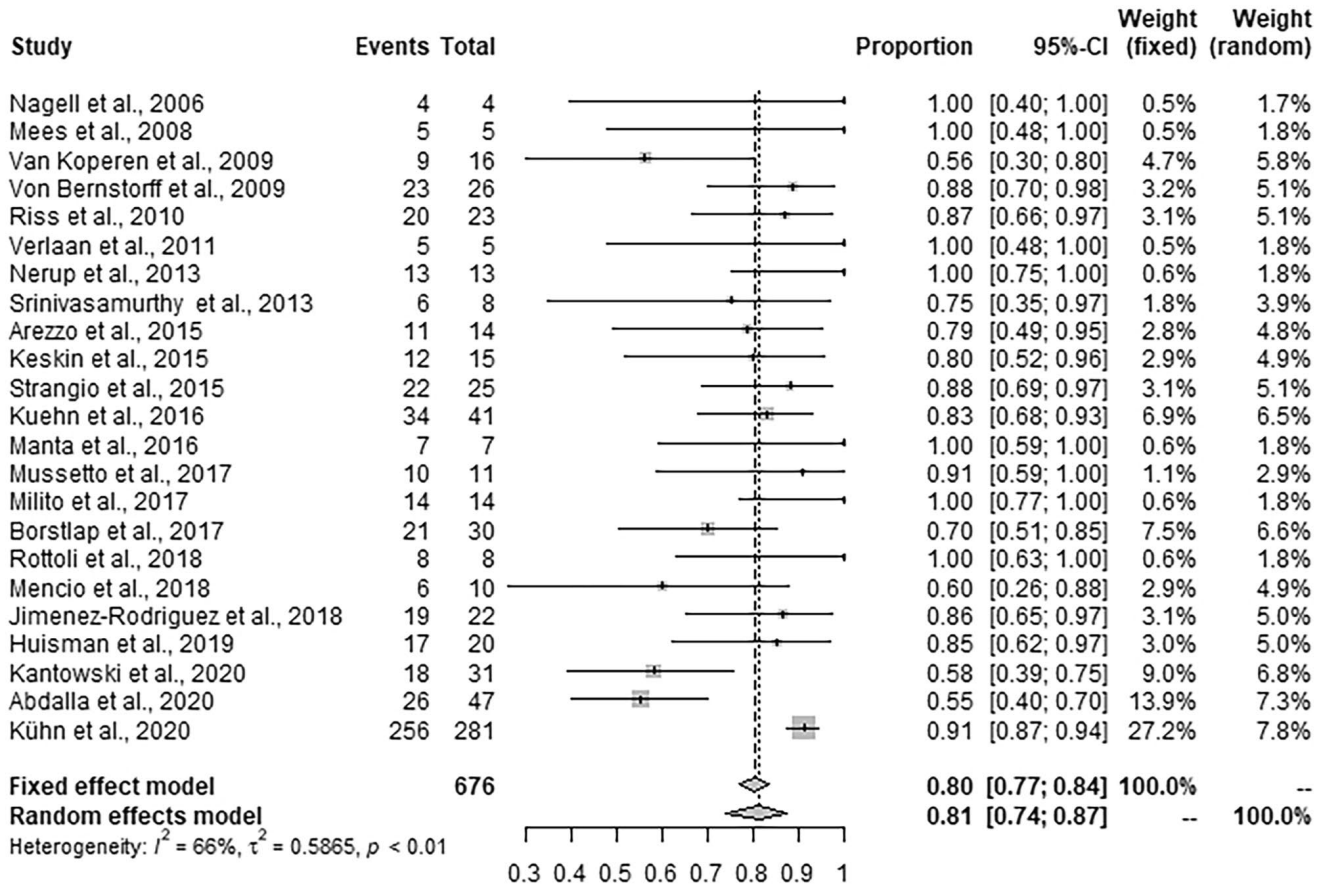
Forest plot for success rate of EVT across the studies. Success rates are shown with 95% CI

In 566 out of 676 patients treated with EVT, treatment success was reported. Random effects meta-analysis showed that the weighted mean success rate of EVT was 81.4% (95% CI: 74.0–87.1%, *I*^2^ = 66.3%) (Fig. 3).

### Fecal diversion

**Fig. 4:**
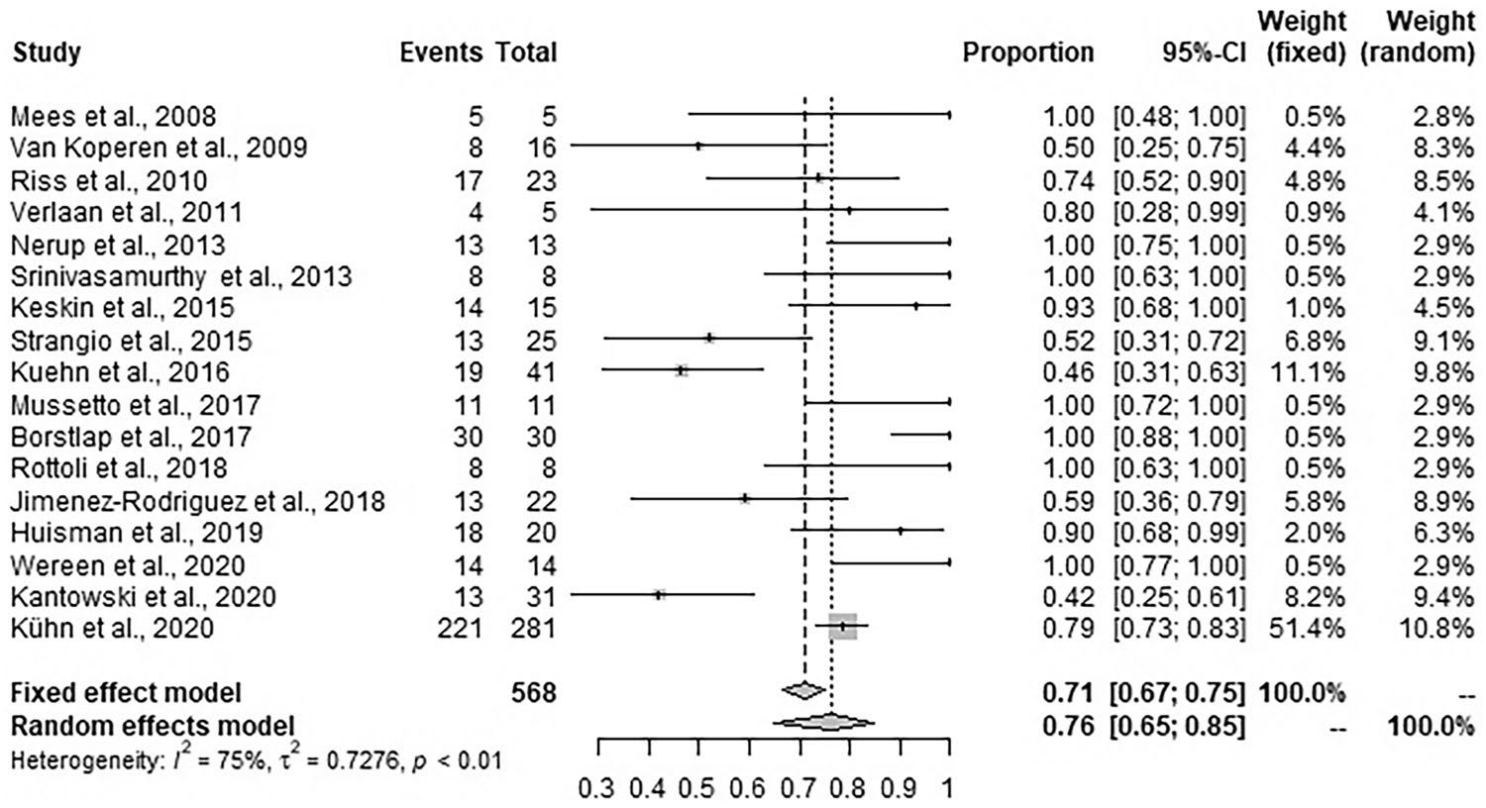
Forest plot for any kind of diverting ostomy during EVT across the studies. Diverting ostomy rates are shown with 95% CI

In 429 of 568 patients, a fecal diversion with any kind of ostomy was reported. Random effects meta-analysis showed that the weighted mean fecal diversion rate during EVT was 76.4% (95% CI: 64.9–85.0%, *I*^2^ = 74.6%) (Fig. 4).

### Stoma reversal rate

**Fig. 5:**
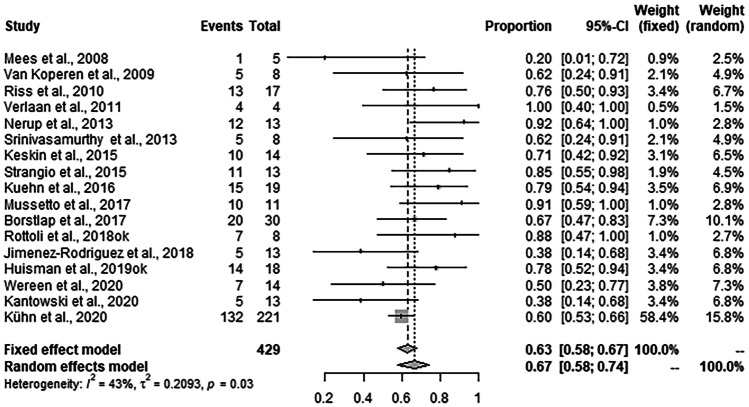
Forrest plot for stoma reversal rate after EVT across the studies. Stoma reversal rates are shown with 95% CI

Ostomy closure was achieved in 276 of 429 patients. Random effects meta-analysis showed a weighted mean ostomy reversal rate after EVT of 66.7% (95% CI: 58.0–74.4%, *I*^2^ = 43.4%) (Fig. 5). Overall 415 of 568 reported patients did not need a permanent ostomy (76.1%), as is another 139 of 568 patients, no ostomy was necessary at all.

### EVT-associated morbidity

**Fig. 6:**
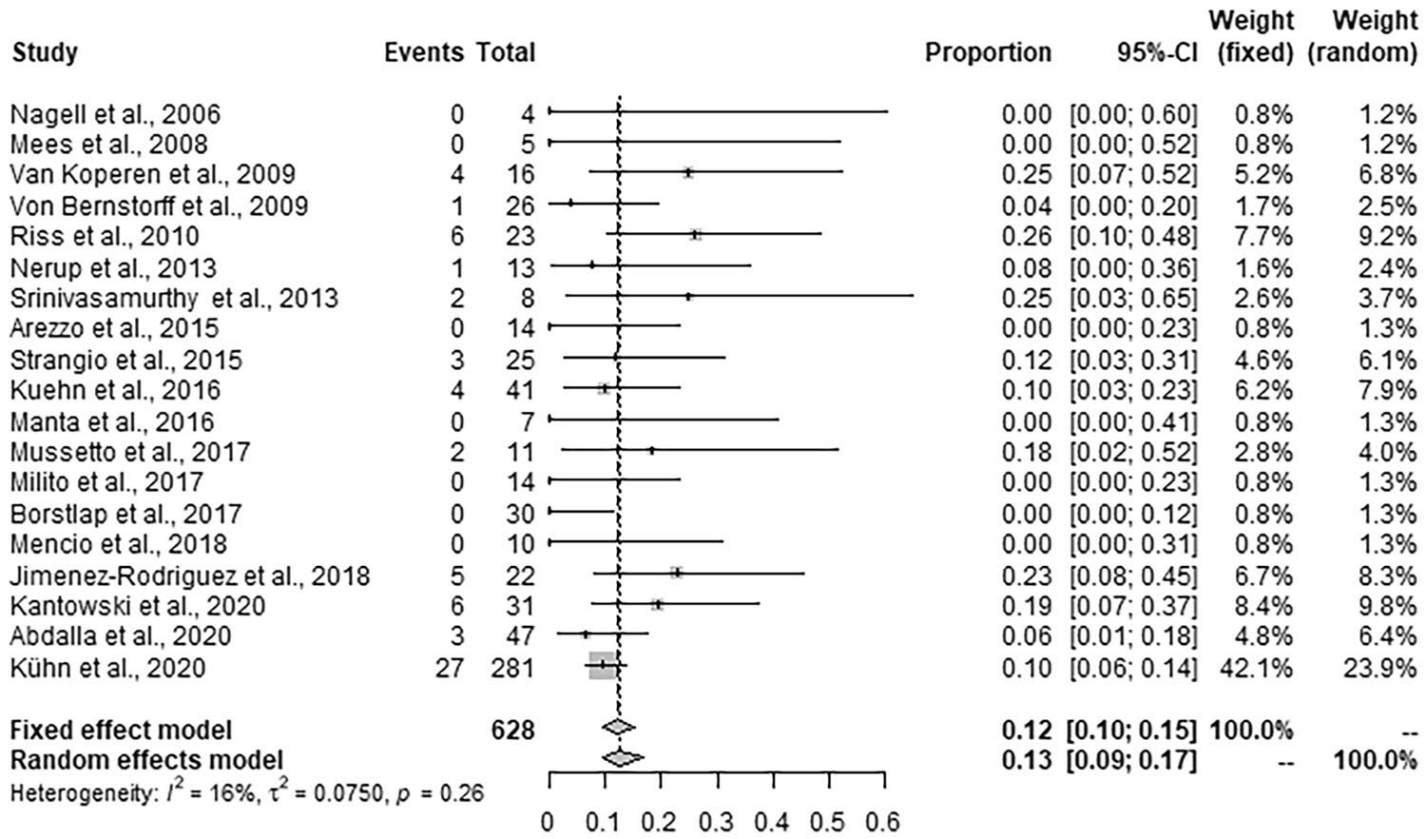
Forrest plot for EVT-associated complication rates across the studies. Complication rates are shown with 95% CI

Complications occurred in 64 of 628 patients. The pooled rate from the fixed effects model was 12.1% (95% CI: 9.7–15.2%]. Due to the homogeneity of the study results (*I*^2^ = 16.0%), the pooled rate from the random effects model was virtually identical (12.7%, 95% CI: 9.5–16.8%) (Fig. 6)**.** Anastomotic stenosis accounted for the most frequent complication after EVT in *n* = 24 (0 up to 18.2% [18]) reported cases, followed by fistulas n = 17 (0 up to 28.6% [24]), abscess formation and chronic sinus (presacral abscess persisting > 12 months) in *n* = 15 (0 up to 21.7% [11]), and bleeding complications *n* = 9 (0 up to 9.7% [25]). There was no reported mortality related to EVT [7–29, 37].

### EVT failure

In 110 of 676 cases, a failure of EVT was reported. Reported reasons for failure of EVT are summarized in Table 2.

**Table 2:** Reported reasons for the failure of EVT

**Reason of failure**	**N (% of 676 cases)**
Non-responder (insufficient granulation tissue/progressive pelvic sepsis/persistent chronic sinus/AL recurrence)	*N* = 80 (11.8%) [7, 9–11, 13–16, 18, 20, 23, 25–27, 37]
Death due to comorbitities unrelated to EVT	*N* = 10 (1.5%) [7, 25, 27, 37]
Technical problems	*N* = 6 (0.9%) [7, 13, 25]
Bleeding	*N* = 4 (0.6%) [7, 9, 15, 25]
Fistulae	*N* = 5 (0.7%) [10, 16, 25]
Lack of compliance/patient’s wish	*N* = 4 (0.6%) [14, 27, 37]
Severe pain	*N* = 1 (0.1%) [9]

## Discussion

EVT was invented and introduced into clinical practice approximately 20 years ago and is currently applied for upper and lower gastrointestinal defects in over 40 countries [2]. However, still no data from RCTs comparing its outcome to any other endoscopic or surgical approach is available. The current systematic review offers the best available evidence for its use in the management of colorectal defects.

Colorectal defects in rectal cancer surgery, surgery for diverticular disease, or traumatic rectal injury are severe medical conditions and mostly difficult to treat with relevant risk for further morbidity and even mortality [4, 38]. With a weighted mean success rate of 81% in this meta-analysis, EVT is a promising, interventional treatment option which is thought to prevent redo-surgery including emergency discontinuity resection in the majority of patients. Although the cumulative experience is still limited and our analysis lacks a control cohort, EVT can be considered one treatment option of the abovementioned conditions.

A systematic review of the risk factors for EVT was performed. Only a very few studies reported on predictors for success or failure for EVT: Von Bernstoff et al. reported that healing took significantly longer when patients received neoadjuvant pretreatment with 31.6 days versus 12.3 days (*p* < 0.001) [10]. Abdalla et al. found female sex (*p* = 0.033) to be associated with EVT success. Furthermore, EVT duration of 21 days or less was associated with a higher rate of success (*p* = 0.017) undergoing primary EVT with 73% compared to salvage with 33% (*p* = 0.006) and when EVT was initiated within 15 days with 72.4% compared to more than 15 days with 27.8% after the diagnosis of AL (*p* = 0.003) [26]. This was supported by the study of Kühn et al. where the risk factors for EVT failure were multi-visceral resection (*p* = 0.037) and surgical revision after primary surgery (*p* = 0.009) [27]. Furthermore, Jimenez-Rodriguez et al. found better results in patients who underwent earlier EVT < 6 weeks after primary surgery compared to a later start (*p* = 0.041) [22].

Because in previous reports it was concluded that bowel diversion is necessary for the effectiveness of EVT in case of AL [6], we examined the number of any kind of diverting ostomies during EVT in the included studies. Surprisingly, we found a weighted mean fecal diversion rate of only 76%. The exact reasons are not described in the underlying data, but most likely in some cases with extraluminal defects or perianastomtic cavities EVT treatment can be successfully performed without diverting ostomy. If the sponge can mainly be placed in the extraluminal defect being accessed transanally through the anastomatic leak, this ensures free luminal passage for the bowel content, whereas in cases the sponge completely obstructs the rectal lumen, EVT is unlikely to be successful [39].

Ostomy reversal was finally possible in only 63% of cases, which is in accordance with the other available data reporting of ostomy reversal rates of 30–40% following AL [40]. Of note, in this series, also rectal stump insufficiency following Hartmann’s procedure was included. Therefore, the ostomy reversal rate should not be considered as a main indicator for the effectiveness of EVT, since restoration of bowel continuity (Hartmann reversal) is usually performed in only about 25–47% of cases even without Hartmann stump insufficiency [41, 42]. On the other hand, as another 139 patients did not need an ostomy at all, 76.1% of patients were ostomy free in further clinical course (415/568). A recently published meta-analysis only including EVT in cases of AL and excluding discontinuity resections reported an ostomy reversal rate of 73% (95% CI: 62–84%) [31]—-although this might represent the subgroup with a less severe clinical problem.

Despite the promising results, the possible complications of EVT should be kept in mind. Considering that these mostly seriously ill patients have a median weighted complication rate of only 12%, EVT has an acceptable safety profile. In this meta-analysis, anastomotic stenosis represented the majority of EVT-associated complications, occurring in a total of 24 patients [7, 11, 12, 18, 22, 27]. As even the rate of anastomotic stenosis following rectal resection was reported to be 22% [43]. As shown in the study by Weidenhagen et al. all stenoses could be successfully treated with an average of six balloon dilations, and an overall ostomy closure rate of 88% was achieved [2]. Other reported complications are fistulas in *n* = 17 [13, 16, 24, 25, 27], abscesses in *n* = 15 [9, 11, 16, 22, 23] and bleeding in *n* = 9 cases [7, 9, 25, 27] in total.

The duration of therapy is one of the major concerns regarding EVT with a weighted treatment duration of 23.4 days (with a range of up to 258 days). Such a long treatment duration, however, may be limited to single cases. One advantage of EVT treatment compared to surgical alternatives is the possibility to transfer a patient into an outpatient setting during treatment [27].

This systematic review is limited by the sparse—and overall quality of available data on EVT. Only data from few prospective studies and no RCT have yet been reported. Most of the included manuscripts are based on a small number of patients. It should also be emphasized that the largest cohort included in this study was published from our institution, which could possibly lead to confirmation bias. The use of EVT in this analysis was not standardized as it was applied to a variety of indications using different techniques. Due to this clinical heterogeneity and the quality of available data, there is a significant heterogeneity among the studies and their background. A relevant selection bias regarding the primary outcome parameter of EVT treatment success needs to be addressed since a complete anastomotic dehiscence with generalized peritonitis will not be treated by EVT, but surgically, and, therefore, is not reported in the included data. The high success rate of about 80% can only be applied to the cases with an extraperitoneal defect without a complete circular necrosis. Therefore, in the treatment of colorectal defects, patients must be selected for the appropriate treatment concept. EVT appears to function well in patients with extraperitoneal colorectal defects in combination with a diverting stoma and without signs of a generalized peritonitis. In these selected cases, EVT is characterized by a good treatment success and moderate complication rates.

Regarding long-term incidence of complications, but also oncological outcome, future prospective, comparative studies with large sample sizes are urgently needed.

## Conclusion

This systematic review and meta-analysis is the largest body of evidence currently collected on EVT. Based on the sparse data, it shows that EVT is a feasible treatment option with manageable risk for selected patients with colorectal leaks.

## References

[1] Peinemann F (2017). Negative pressure wound therapy: randomised controlled trials from 2000 to 2015. Zentralbl Chir.

[2] Weidenhagen R, Gruetzner KU, Wiecken T, Spelsberg F, Jauch K-W (2008). Endoscopic vacuum-assisted closure of anastomotic leakage following anterior resection of the rectum: a new method. Surg Endosc.

[3] Kuehn F, Schiffmann L, Janisch F, Schwandner F, Alsfasser G, Gock M (2016). Surgical endoscopic vacuum therapy for defects of the upper gastrointestinal tract. J Gastrointest Surg.

[4] Jannasch O, Klinge T, Otto R, Chiapponi C, Udelnow A, Lippert H et al (2015) Risk factors, short and long term outcome of anastomotic leaks in rectal cancer. Oncotarget [Internet]. 2015 Nov 3 [cited 2019 Oct 20];6(34). Available from: http://www.oncotarget.com/fulltext/5170 10.18632/oncotarget.5170 PMC4742217 26392333

[5] Shalaby M, Emile S, Elfeki H, Sakr A, Wexner SD, Sileri P (2019). Systematic review of endoluminal vacuum-assisted therapy as salvage treatment for rectal anastomotic leakage. BJS Open.

[6] Popivanov GI, Mutafchiyski VM, Cirocchi R, Chipeva SD, Vasilev VV, Kjossev KTs et al (2020) Endoluminal negative pressure therapy in colorectal anastomotic leaks. Colorectal Dis 22(3):243–53 10.1111/codi.14754 31274227

[7] Kuehn F, Janisch F, Schwandner F, Alsfasser G, Schiffmann L, Gock M (2016). Endoscopic vacuum therapy in colorectal surgery. J Gastrointest Surg.

[8] Nagell CF, Holte K (2006). Treatment of anastomotic leakage after rectal resection with transrectal vacuum-assisted drainage (VAC): a method for rapid control of pelvic sepsis and healing. Int J Colorectal Dis.

[9] van Koperen PJ, van Berge Henegouwen MI, Rosman C, Bakker CM, Heres P, Slors JFM (2009). The Dutch multicenter experience of the endo-sponge treatment for anastomotic leakage after colorectal surgery. Surg Endosc.

[10] von Bernstorff W, Glitsch A, Schreiber A, Partecke LI, Heidecke CD (2009). ETVARD (endoscopic transanal vacuum-assisted rectal drainage) leads to complete but delayed closure of extraperitoneal rectal anastomotic leakage cavities following neoadjuvant radiochemotherapy. Int J Colorectal Dis.

[11] Riss S, Stift A, Kienbacher C, Dauser B, Haunold I, Kriwanek S (2010). Recurrent abscess after primary successful endo-sponge treatment of anastomotic leakage following rectal surgery. WJG.

[12] Nerup N, Johansen JL, Alkhefagie GAA, Maina P, Jensen KH (2013) Promising results after endoscopic vacuum treatment of anastomotic leakage following resection of rectal cancer with ileostomy. 4 23651714

[13] Srinivasamurthy D, Wood C, Slater R, Garner J (2013). An initial experience using transanal vacuum therapy in pelvic anastomotic leakage. Tech Coloproctol.

[14] Arezzo A, Verra M, Passera R, Bullano A, Rapetti L, Morino M (2015). Long-term efficacy of endoscopic vacuum therapy for the treatment of colorectal anastomotic leaks. Dig Liver Dis.

[15] Keskin M, Bayram O, Bulut T, Balik E (2015). Effectiveness of endoluminal vacuum-assisted closure therapy (endosponge) for the treatment of pelvic anastomotic leakage after colorectal surgery. Surgical Laparoscopy, Endoscopy & Percutaneous Techniques.

[16] Strangio G, Zullo A, Ferrara EC, Anderloni A, Carlino A, Jovani M (2015). Endo-sponge therapy for management of anastomotic leakages after colorectal surgery: a case series and review of literature. Dig Liver Dis.

[17] Manta R, Caruso A, Cellini C, Sica M, Zullo A, Mirante VG (2016). Endoscopic management of patients with post-surgical leaks involving the gastrointestinal tract: a large case series. United European Gastroenterol j.

[18] Mussetto A, Arena R, Buzzi A, Fuccio L, Dari S, Brancaccio ML et al (2017) Long-term efficacy of vacuum-assisted therapy (Endo-SPONGE®) in large anastomotic leakages following anterior rectal resection. aog [Internet]. 2017 [cited 2021 Mar 21]; Available from: http://www.annalsgastro.gr/files/journals/1/earlyview/2017/ev-09-2017-06-AG3133-0194.pdf 10.20524/aog.2017.0194 PMC5670284 29118559

[19] Milito G, Lisi G, Venditti D, Campanelli M, Aronadio E (2017) Endoluminal vacuum therapy as treatment for anastomotic colorectal leakage. General Surg 30:7 28072899

[20] Borstlap WAA, Musters GD, Stassen LPS, van Westreenen HL, Hess D, van Dieren S (2018). Vacuum-assisted early transanal closure of leaking low colorectal anastomoses: the CLEAN study. Surg Endosc.

[21] Rottoli M, Di Simone MP, Vallicelli C, Vittori L, Liguori G, Boschi L (2018). Endoluminal vacuum-assisted therapy as treatment for anastomotic leak after ileal pouch–anal anastomosis: a pilot study. Tech Coloproctol.

[22] Jimenez-Rodriguez RM, Araujo-Miguez A, Sobrino-Rodriguez S, Heller F, Díaz-Pavon JM, Bozada Garcia JM (2018). A new perspective on vacuum-assisted closure for the treatment of anastomotic leak following low anterior resection for rectal cancer, is it worthy?. Surg Innov.

[23] Huisman JF, van Westreenen HL, van der Wouden EJ, Vasen HFA, de Graaf EJR, Doornebosch PG (2019). Effectiveness of endosponge therapy for the management of presacral abscesses following rectal surgery. Tech Coloproctol.

[24] Weréen A, Dahlberg M, Heinius G, Pieniowski E, Saraste D, Eklöv K (2020). Long-term results after anastomotic leakage following rectal cancer surgery: a comparison of treatment with endo-sponge and transanal irrigation. Dig Surg.

[25] Kantowski M, Kunze A, Bellon E, Rösch T, Settmacher U, Tachezy M (2020). Improved colorectal anastomotic leakage healing by transanal rinsing treatment after endoscopic vacuum therapy using a novel patient-applied rinsing catheter. Int J Colorectal Dis.

[26] Abdalla S, Cotte E, Epin A, Karoui M, Lefevre JH, Berger A (2020). Short-term and long-term outcome of endoluminal vacuum therapy for colorectal or coloanal anastomotic leakage: results of a nationwide multicenter cohort study from the French GRECCAR Group. Dis Colon Rectum.

[27] Kühn F, Wirth U, Zimmermann J, Beger N, Hasenhütl SM, Drefs M et al (2020) Endoscopic vacuum therapy for in- and outpatient treatment of colorectal defects. Surg Endosc [Internet]. 2020 Dec 1 [cited 2021 Feb 11]; Available from: http://link.springer.com/10.1007/s00464-020-08172-5 10.1007/s00464-020-08172-5 PMC8599392 33259019

[28] Mees ST, Palmes D, Mennigen R, Senninger N, Haier J, Bruewer M (2008) Endo-vacuum Assisted closure treatment for rectal anastomotic insufficiency. Dis colon & rectum 51(4):404–10 10.1007/s10350-007-9141-z 18197452

[29] Verlaan T, Bartels SAL, van Berge Henegouwen MI, Tanis PJ, Fockens P, Bemelman WA (2011). Early, minimally invasive closure of anastomotic leaks: a new concept: early, minimally invasive closure of anastomotic leaks. Colorectal Dis.

[30] Srinivasamurthy D, Wood C, Slater R, Garner J (2013) An initial experience using transanal vacuum therapy in pelvic anastomotic leakage. Tech Coloproctol 7 10.1007/s10151-012-0911-9 23111399

[31] Popivanov GI, Mutafchiyski VM, Cirocchi R, Chipeva SD, Vasilev VV, Kjossev KT et al (2019) Endoluminal negative pressure therapy in colorectal anastomotic leaks. 11 10.1111/codi.14754 31274227

[32] Sharp G, Steffens D, Koh CE (2021). Evidence of negative pressure therapy for anastomotic leak: a systematic review. ANZ J Surg.

[33] Kühn F, Janisch F, Schwandner F, Gock M, Wedermann N, Witte M (2020). Comparison between endoscopic vacuum therapy and conventional treatment for leakage after rectal resection. World J Surg.

[34] Shamseer L, Moher D, Clarke M, Ghersi D, Liberati A, Petticrew M et al (2015) Preferred reporting items for systematic review and meta-analysis protocols (PRISMA-P) 2015: elaboration and explanation. BMJ 349(jan02 1):g7647–g7647 10.1136/bmj.g7647 25555855

[35] R Core Team (2020) R: A language and environment for statistical computing. [Internet]. Vienna, Austria: R Foundation for Statistical Computing; 2020. Available from: https://www.R-project.org/

[36] Balduzzi S, Rücker G, Schwarzer G (2019). How to perform a meta-analysis with R: a practical tutorial. Evid Based Mental Health.

[37] Mencio MA, Ontiveros E, Burdick JS, Leeds SG (2018). Use of a novel technique to manage gastrointestinal leaks with endoluminal negative pressure: a single institution experience. Surg Endosc.

[38] Snijders HS, Wouters MWJM, van Leersum NJ, Kolfschoten NE, Henneman D, de Vries AC (2012). Meta-analysis of the risk for anastomotic leakage, the postoperative mortality caused by leakage in relation to the overall postoperative mortality. European Journal of Surgical Oncology (EJSO).

[39] Kühn F, Hasenhütl SM, Hoffman F (2021) Endoscopic vacuum therapy for left-sided colorectal anastomotic leak without fecal diversion. Dis Colon Rectum (Forthcoming) 10.1097/DCR.0000000000001959 34775405

[40] Holmgren K, Kverneng Hultberg D, Haapamäki MM, Matthiessen P, Rutegård J, Rutegård M (2017). High stoma prevalence and stoma reversal complications following anterior resection for rectal cancer: a population-based multicentre study. Colorectal Dis.

[41] Vermeulen J, Coene PPLO, Van Hout NM, van der Harst E, Gosselink MP, Mannaerts GHH (2009). Restoration of bowel continuity after surgery for acute perforated diverticulitis: should Hartmann’s procedure be considered a one-stage procedure?. Colorectal Dis.

[42] Hallam S, Mothe B, Tirumulaju R (2018) Hartmann’s procedure, reversal and rate of stoma-free survival. Annals 100(4):301–7 10.1308/rcsann.2018.0006 PMC5958852 29484943

[43] Jain D, Sandhu N, Singhal S (2017) Endoscopic electrocautery incision therapy for benign lower gastrointestinal tract anastomotic strictures. Aog 30:473–85 10.20524/aog.2017.0163 PMC5566767 28845102

